# Myocardial Stress Biomarkers and Inflammatory Indices in Cattle With Bovine Respiratory Disease Complex

**DOI:** 10.1002/vms3.71096

**Published:** 2026-07-20

**Authors:** Şükrü Değirmençay, Reyhane Bayat

**Affiliations:** ^1^ Department of Internal Medicine Faculty of Veterinary Medicine Atatürk University Erzurum Turkey

**Keywords:** bovine respiratory disease, heart‐type fatty acid‐binding protein, inflammatory indices, lymphocyte‐to‐monocyte ratio, myocardial stress

## Abstract

**Background:**

Bovine respiratory disease complex (BRDC) is primarily considered a pulmonary disorder; however, hypoxia and systemic inflammation associated with this condition may also impose cardiovascular stress.

**Objectives:**

This exploratory study investigated myocardial stress biomarkers in cattle with BRDC and evaluated their relationships with haematological inflammatory indices and ratio‐derived enzyme patterns reflecting tissue injury dynamics.

**Methods:**

Twenty‐four Simmental crossbred cattle aged 6–10 months were included, comprising 16 BRDC‐affected animals and 8 clinically healthy controls. Myocardial stress biomarkers, including heart‐type fatty acid‐binding protein (H‐FABP), creatine kinase‐MB (CK‐MB), creatine kinase (CK), aspartate aminotransferase (AST) and lactate dehydrogenase (LDH), were measured. Haematological indices and ratio‐derived parameters, including the lymphocyte‐to‐monocyte ratio (LMR), AST/CK and CK‐MB/H‐FABP, were calculated. Group comparisons, correlation analyses, receiver operating characteristic analysis and multivariable linear regression were performed.

**Results:**

Compared with controls, BRDC cattle showed significantly higher WBC, LYM, LMR, H‐FABP, CK‐MB, CK, AST, LDH, AST/CK, CK‐MB/H‐FABP, heart rate, respiratory rate and rectal temperature, whereas MCH and MCHC were lower (*p* < 0.05). For example, H‐FABP concentrations were higher in BRDC cattle (0.65 ± 0.09 vs. 0.34 ± 0.07 ng/mL), and heart rate was markedly increased (146 [106–184] vs. 79 [60–88] beats/min). H‐FABP correlated positively with inflammatory and physiological parameters and strongly with heart rate. Multivariable linear regression identified RBC as an independent factor associated with H‐FABP concentrations (*p* < 0.001), whereas LMR was not independently associated with H‐FABP after adjustment (*p* = 0.087; adjusted *R* ^2^ = 0.837). ROC analysis indicated preliminary discriminatory capacity for several biomarkers, although these findings should be interpreted cautiously given the limited sample size and potential overfitting.

**Conclusions:**

BRDC may be accompanied by elevations in myocardial stress biomarkers that parallel systemic inflammatory activation and physiological stress. Integrated evaluation of myocardial stress biomarkers and inflammatory indices may offer a potentially useful exploratory framework for characterizing cardiopulmonary stress responses in BRDC; however, further validation in larger cohorts is required.

## Graphical abstract

Bovine respiratory disease complex may involve systemic cardiopulmonary stress beyond primary lung pathology. Combined elevation of myocardial stress biomarkers (H‐FABP, CK‐MB) and inflammatory indices (LMR) suggests that integrated biomarker assessment may help identify cardiopulmonary stress responses in BRDC‐affected cattle under field conditions.

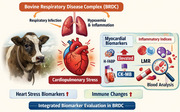

## 1 Introduction

Bovine respiratory disease complex (BRDC) is a multifactorial syndrome driven by the interaction of stress, viral agents and bacterial pathogens and represents one of the leading causes of morbidity and economic loss in young cattle (Patel et al. 2017). Although respiratory pathology predominates, increasing evidence suggests that BRDC may also exert secondary effects on the cardiovascular system through mechanisms involving hypoxic stress, systemic inflammation and altered haemodynamics (Anderson et al. 2017; Değirmençay 2023).

Elevated circulating concentrations of cardiac‐associated biomarkers, including heart‐type fatty acid‐binding protein (H‐FABP), creatine kinase‐MB (CK‐MB), lactate dehydrogenase (LDH) and aspartate aminotransferase (AST), have been reported in BRDC‐affected calves, suggesting myocardial stress in addition to pulmonary involvement (Anderson et al. 2017; Değirmençay 2023). H‐FABP is a highly sensitive early‐release biomarker of myocardial injury that can increase in circulation within the first hours following myocardial damage and has been used in human medicine for early detection of acute myocardial infarction. In comparative studies, H‐FABP has demonstrated high diagnostic sensitivity and specificity for early myocardial injury and may increase earlier than conventional cardiac biomarkers such as CK‐MB and troponins (Gerede et al. 2015). However, interpretation of these biomarkers is complicated by their potential release from non‐cardiac tissues during systemic inflammation, hypoxia, or generalized tissue injury (Dufour 1988; Wu et al. 2020). Therefore, evaluation based solely on single‐marker elevation may not sufficiently differentiate myocardial stress from concurrent skeletal muscle or hepatic involvement.

Ratio‐derived indices may provide additional insight into tissue injury patterns. The AST/CK ratio has been used to aid differentiation between cardiac and skeletal muscle sources of enzyme elevation by leveraging differences in tissue distribution (Dufour 1988; Swain et al. 1993). Similarly, the CK‐MB/H‐FABP ratio may reflect relative release dynamics of enzymes with differing tissue specificity and kinetic properties (Rezar et al. 2020; Wu et al. 2020). A pattern‐based interpretation integrating multiple biomarkers and derived ratios may therefore enhance understanding of cardiopulmonary interactions in BRDC.

In parallel, haematological indices derived from complete blood counts (CBCs) have emerged as accessible indicators of systemic inflammatory burden. The lymphocyte‐to‐monocyte ratio (LMR) has been associated with disease severity and cardiovascular prognosis in human medicine (Chen et al. 2020; Kiris et al. 2017; Silva et al. 2015; Zhao et al. 2022). In inflammatory conditions, alterations in leukocyte subpopulations may reflect the magnitude of immune activation and cytokine‐mediated responses (Ceciliani et al. 2012). In the context of BRDC, systemic inflammation and hypoxemia may together influence myocardial oxygen balance, potentially linking inflammatory burden to elevations in myocardial stress biomarkers.

We hypothesized that (1) BRDC is associated with increased circulating myocardial stress biomarkers; (2) ratio‐based enzyme indices may reflect differential tissue injury patterns; and (3) systemic inflammatory burden, as reflected by haematological indices, is associated with elevations in myocardial biomarkers. Accordingly, the objectives of this study were to characterize biochemical and haematological alterations in BRDC‐affected cattle and to explore the relationship between inflammatory burden and myocardial stress biomarkers using a ratio‐based analytical approach. To our knowledge, this is among the first studies integrating myocardial stress biomarkers with inflammatory haematological indices to explore cardiopulmonary interactions in cattle with BRDC. This integrative approach may improve interpretation of systemic physiological stress responses associated with respiratory disease in cattle. From a clinical perspective, improved recognition of cardiopulmonary stress patterns in BRDC may assist veterinarians in identifying systemic disease severity under field conditions where advanced cardiac diagnostics are rarely available.

## 2 Materials and Methods

This research was conducted in compliance with the ethical guidelines approved by Atatürk University (protocol number: 2025/08, decision number: 147). Written informed consent was obtained from the owner of each animal prior to sample collection.

### 2.1 Animals and Protocol Design

The study population consisted of 24 Simmental crossbred cattle, aged between 6 and 10 months, including both males and females. Based on clinical assessment, clinical scoring system and CBC results, the animals were categorized into two groups: BRDC‐affected (*n* = 16) and healthy controls (*n* = 8). During the clinical evaluation, rectal temperature (RT), heart rate (HR) and respiratory rate (RR) were recorded for each calf. Cattle that scored 5 or above according to the scoring criteria proposed by Love et al. (2014), specifically the third clinical scoring system described in that study, were classified as BRDC cases. This system includes spontaneous coughing (2 points), nasal discharge (any type, 4 points), ocular discharge (2 points), ear and head carriage abnormalities (5 points), fever ≥ 39.2°C (2 points) and abnormal respiratory quality (2 points), with a diagnostic threshold of ≥ 5 points for BRDC classification. According to this system (Table 1), animals exhibiting abnormal ear or head posture, those with nasal discharge plus one additional clinical sign, or those presenting any three clinical signs were considered positive for BRDC. Diagnosis was based on clinical findings and established scoring criteria; advanced imaging or pathogen‐specific diagnostic testing was not performed. Due to the exploratory nature of the study and field availability of cases, a formal a priori sample size calculation was not performed. Therefore, given the limited sample size and exploratory design, this study should be considered a pilot investigation.

**TABLE 1:** Clinical scoring system for bovine respiratory disease complex (BRDC) based on the third model described by Love et al. (2014).

Clinical sign	Level	Score
Cough	None or induced cough	0
	Spontaneous cough	2
Nasal discharge	None	0
	Any	4
Ocular discharge	None	0
	Any	2
Ear position	Normal, ear flick or head shake	0
	Ear droop or head tilt	5
Rectal temperature	< 39.2°C	0
	≥ 39.2°C	2
Abnormal respiration	Absent	0
	Present	2

*Note*: BRDC diagnosis was defined as a total score ≥ 5 according to the third scoring system of Love et al. (2014).

### 2.2 Blood Sampling

Venous blood was collected from the external jugular vein of each calf into EDTA tubes (Vacutainer, K2E 3.6 mg, BD, UK) for haematological testing and into gel‐containing tubes (Vacutainer, BD, UK) for biochemical analysis. The gel tubes were maintained at room temperature and centrifuged at 3000 rpm for 10 min. Separated serum samples were aliquoted and stored at –80°C until biochemical measurements were performed. Haematological assessments were completed immediately after collection. Laboratory analyses were performed by personnel blinded to group allocation.

### 2.3 Haematological Analyses

White blood cell (WBC), lymphocyte (LYM), monocyte (MON), neutrophil (NEU), red blood cell (RBC), haemoglobin (HGB), haematocrit (HCT), mean corpuscular volume (MCV), mean corpuscular haemoglobin (MCH), mean corpuscular haemoglobin concentration (MCHC) and platelet (PLT) levels of the cattle were determined by a haematology analyser (Abacus Junior Vet5, Hungary). Haematological indices were calculated from leukocyte subpopulations, including the LMR and neutrophil‐to‐lymphocyte ratio (NLR).

### 2.4 Biochemical Analyses

Serum H‐FABP levels were quantified using commercially available, bovine‐specific ELISA kits (Sunred Biological Technology, Shanghai, China; Catalogue No: 201‐04‐2718), following the manufacturer's instructions. The assay sensitivity was reported as 0.074 ng/mL, with a measurement range of 0.08–20 ng/mL. The intra‐assay and inter‐assay coefficients of variation (CV) were < 10% and < 12%, respectively, as provided by the manufacturer. Although the assay is designed for bovine samples, full analytical validation in bovine serum (e.g., spike‐recovery, linearity‐of‐dilution and matrix effects) was not independently verified in the present study and should be considered a limitation. Serum activities of CK, CK‐MB, LDH and AST were measured using a fully automated biochemical analyser (Beckman Coulter AU5800, USA) with standardized commercial reagent kits. AST/CK and CK‐MB/H‐FABP ratios were calculated as follows: AST/CK = AST/CK activity ratio; CK‐MB/H‐FABP = CK‐MB concentration to H‐FABP concentration ratio.

### 2.5 Statistical Analyses

Statistical analyses were conducted using SPSS software (version 25.0; SPSS Inc., Chicago, IL, USA). The Shapiro–Wilk test was employed to evaluate the normality of data distribution between the BRDC and healthy groups. For variables demonstrating normal distribution (WBC, LYM, NEU, PLT, H‐FABP, CK‐MB, CK‐MB/H‐FABP and RR), comparisons were made using the Independent Samples *t*‐test. Variables not conforming to a normal distribution (MON, NLR, LMR, RBC, HGB, HCT, MCV, MCH, MCHC, CK, LDH, AST, AST/CK, RT and HR) were analysed using the Mann–Whitney *U* test. Associations between parameters were evaluated using Spearman's rank correlation analysis. Correlation coefficients were interpreted as follows: weak (0.10–0.39), moderate (0.40–0.69), strong (0.70–0.89) and very strong (0.90–1.00) (Mukaka 2012). Receiver operating characteristic (ROC) curve analysis was performed to evaluate the discriminatory performance of selected biomarkers and ratio‐derived indices between BRDC‐affected and control cattle. Sensitivity, specificity and area under the curve (AUC) were calculated for selected biomarkers and ratio‐derived indices. The AUC values were classified as follows: fail (0.5 ≤ AUC < 0.6), poor (0.6 ≤ AUC < 0.70), fair (0.7 ≤ AUC < 0.8), good (0.8 ≤ AUC < 0.9) and excellent (0.9 ≤ AUC) (Nahm 2022). To evaluate independent associations, multivariable linear regression analysis was performed with H‐FABP as the dependent variable and LMR and RBC as independent variables. Variables included in the multivariable model were selected based on biological plausibility and significant associations observed in univariable analyses to minimize overfitting given the limited sample size. Collinearity diagnostics were assessed using variance inflation factors (VIF). Given the exploratory nature and multiple statistical comparisons performed, no formal adjustment for multiplicity (e.g., false discovery rate) was applied; therefore, results should be interpreted cautiously due to increased risk of Type I error. A significance level of *p* < 0.05 was considered statistically significant. Parametric data were expressed as mean ± standard deviation (SD), whereas nonparametric data were presented as median (minimum–maximum). The complete raw dataset, including individual animal clinical scores and laboratory measurements, is provided as Table S1 to support transparency and enable future re‐analysis.

## 3 Results

### 3.1 Clinical Findings

Common clinical manifestations observed in BRDC‐affected cattle included reduced appetite, elevated body temperature, tachypnea, dyspnea, coughing, nasal and ocular discharge, pale to moderately cyanotic mucous membranes and tachycardia. Compared to the healthy control group, the BRDC group exhibited significantly higher RT (*p* = 0.037), HR (*p* < 0.001) and RR (*p* < 0.001) (RT: 39.46 [38.86–40.7]°C vs. 39.05 [38.4–39.3]°C; HR: 146 [106–184] vs. 79 [60–88] beats/min; RR: 46 ± 5.71 vs. 34 ± 10 breaths/min) (Table 2).

**TABLE 2:** Haematological parameters and vital signs in BRDC‐affected and healthy control cattle.

Parameters	Healthy (*n* = 8)	BRDC (*n* = 16)	*p* value
**WBC (×10^3^/µL)**	6.62 ± 1.50	**8.56 ± 2.16**	**0.033**
**LYM (×10^3^/µL)**	3.42 ± 1.48	**4.71 ± 0.77**	**0.046**
MON (×10^3^/µL)	0.205 (0.07–0.68)	0.09 (0.06–0.35)	0.132
NEU (×10^3^/µL)	2.83 ± 1.46	3.62 ± 1.53	0.236
**RBC (×10^6^/µL)**	5.66 (5.24–8.20)	**11.36 (10.20–13.58)**	**< 0.001**
**HGB (g/dL)**	6.6 (5.4–9.7)	**11.55 (10.5–12.9)**	**< 0.001**
**HCT (%)**	18.67 (15.73–27.37)	**38.64 (34.08–41.41)**	**< 0.001**
MCV (fL)	33 (29–45)	32.5 (30–37)	0.495
**MCH (pg)**	11.6 (9.5–15.2)	**10 (9.4–10.9)**	**0.018**
**MCHC (g/dL)**	34.9 (33.3–36.4)	**30.55 (29.4–31.4)**	**< 0.001**
**PLT (×10^3^/µL)**	309 ± 107	**519 ± 185**	**0.007**
**RT (°C)**	39.05 (38.4–39.3)	**39.46 (38.86–40.7)**	**0.037**
**HR (beats/min)**	79 (60–88)	**146 (106–184)**	**< 0.001**
**RR (breaths/min)**	34 ± 10	**46 ± 5.71**	**< 0.001**

*Note*: Data are presented as mean ± SD and/or median (range).

Abbreviations: HCT, haematocrit; HGB, haemoglobin; HR, heart rate; LYM, lymphocyte; MCH, mean corpuscular haemoglobin; MCHC, mean corpuscular haemoglobin concentration; MCV, mean corpuscular volume; MON, monocyte; NEU, neutrophil; PLT, platelet; RBC, red blood cell; RR, respiratory rate; RT, rectal temperature; WBC, white blood cell.

### 3.2 Haematological Alterations

Haematological analysis revealed significantly elevated WBC, LYM, LMR, RBC, HGB, HCT and PLT levels in the BRDC group compared to healthy controls, whereas MCH and MCHC values were significantly lower (*p* < 0.05) (e.g., WBC: 8.56 ± 2.16 vs. 6.62 ± 1.50 ×10^3^/µL; RBC: 11.36 [10.20–13.58] vs. 5.66 [5.24–8.20] ×10^6^/µL; HCT: 38.64 [34.08–41.41]% vs. 18.67 [15.73–27.37]%) (Table 2).

### 3.3 Biochemical Alterations

The mean H‐FABP and CK‐MB concentrations (*p* < 0.001) and CK‐MB/H‐FABP ratio (*p* = 0.011), as well as median CK (*p* = 0.003), LDH (*p *< 0.001), AST (*p *< 0.001) and AST/CK ratio (*p* = 0.001), were significantly higher in the BRDC group compared with controls (Table 3). For example, H‐FABP concentrations were higher in BRDC cattle (0.65 ± 0.09 vs. 0.34 ± 0.07 ng/mL), and CK‐MB concentrations showed a similar increase (414 ± 94 vs. 135 ± 49 U/L). CK activity was also elevated (318 [167–372] vs. 119 [59–344] U/L).

**TABLE 3:** Myocardial stress biomarkers and haematological ratios in BRDC‐affected and healthy control cattle.

Parameters	Healthy (*n* = 8)	BRDC (*n* = 16)	*p* value
**H‐FABP (ng/mL)**	0.34 ± 0.07	**0.65 ± 0.09**	**< 0.001**
**CK‐MB (U/L)**	135 ± 49	**414 ± 94**	**< 0.001**
**CK (U/L)**	119 (59–344)	**318 (167–372)**	**0.003**
**LDH (U/L)**	490 (278–1259)	**2609 (2087–2793)**	**< 0.001**
**AST (U/L)**	19.5 (10–71)	**111 (71–139)**	**< 0.001**
**AST/CK**	0.18 (0.12–0.37)	**0.38 (0.32–0.49)**	**0.001**
**CK‐MB/H‐FABP**	409 ± 173	**664 ± 228**	**0.011**
NLR	0.85 (0.08–2.05)	0.71 (0.33–1.36)	0.327
**LMR**	14.81 (9.25–32.50)	**45.17 (16.17–75.29)**	**0.001**

*Note*: Data are presented as mean ± SD and/or median (range).

Abbreviations: AST, aspartate aminotransferase; CK, creatine kinase; CK‐MB, creatine kinase myocardial band; H‐FABP, heart type fatty acid binding protein; LDH, lactate dehydrogenase; LMR, lymphocyte‐to‐monocyte ratio; NLR, neutrophil‐to‐lymphocyte ratio.

### 3.4 Associations Between Myocardial Biomarkers and Clinical and Haematological Parameters

A strong positive correlation was identified between HR and H‐FABP (*r* = 0.748, *p* < 0.001), representing the strongest association observed between physiological parameters and myocardial stress biomarkers in the study. HR was moderately positively correlated with CK‐MB (*r* = 0.519, *p* = 0.009), CK (*r* = 0.517, *p* = 0.010), LDH (*r* = 0.490, *p* = 0.015), AST/CK (*r* = 0.556, *p* = 0.005) and AST (*r* = 0.603, *p* = 0.002). RR showed strong correlation with CK (*r* = 0.701, *p* < 0.001), while RR showed moderately positive correlations with CK‐MB (*r* = 0.464, *p* = 0.022), LDH (*r* = 0.483, *p* = 0.017) and AST (*r* = 0.528, *p* = 0.008) (Table 4).

**TABLE 4:** Spearman correlation coefficients between vital parameters and myocardial stress biomarkers in cattle.

Parameters	RT	RR	HR	H‐FABP	CK‐MB	CK	LDH	AST	AST/CK	CK‐MB/H‐FABP
**RT**	1.000	0.485^*^	0.591^**^	0.390	0.425^*^	0.124	0.219	0.226	0.205	0.284
**RR**		1.000	0.629^**^	0.400	**0.464** ^*^	0.701^**^	0.483^*^	0.528^**^	0.180	0.245
**HR**			1.000	**0.748** ^**^	**0.519** ^**^	0.517^**^	0.490^*^	0.603^**^	0.556^**^	0.248

*Note*: Spearman correlation coefficients (*r*).

Abbreviations: AST, aspartate aminotransferase; CK, creatine kinase; CK‐MB, creatine kinase myocardial band; H‐FABP, heart type fatty acid binding protein; HR, heart rate (per min); LDH, lactate dehydrogenase; RR, respiratory rate (per min); RT, rectal temperature.

* *p* < 0.05.

** *p* < 0.01.

Serum H‐FABP concentrations also exhibited moderate positive correlations with inflammatory parameters including WBC (*r* = 0.433, *p* = 0.035), LYM (*r* = 0.471, *p* = 0.020), LMR (*r* = 0.441, *p* = 0.031), CK‐MB (*r* = 0.439, *p* = 0.032) and AST (*r* = 0.502, *p* = 0.012). Strong correlations were observed between H‐FABP and LDH (*r* = 0.726, *p* < 0.001) and AST/CK (*r* = 0.758, *p* < 0.001) (Table 5).

**TABLE 5:** Spearman correlations between leukocyte parameters and myocardial stress biomarkers in cattle.

Parameters	WBC	LYM	LMR	H‐FABP	CK‐MB	CK	LDH	AST	AST/CK	CK‐MB/H‐FABP
**WBC**	1.000	0.706^**^	0.271	**0.433** ^*^	0.157	0.087	0.165	0.161	0.218	0.0050
**LYM**		1.000	0.263	**0.471** ^**^	0.358	0.237	0.302	0.360	0.233	0.319
**LMR**			1.000	**0.441** ^**^	0.528^**^	0.450^*^	0.440^*^	0.403	0.293	0.324
**H‐FABP**				1.000	0.439^*^	0.249	0.726^**^	0.502^*^	0.758^**^	0.030
**CK‐MB**					1.000	0.636^**^	0.722^**^	0.766^**^	0.418^*^	0.878^**^
**CK**						1.000	0.476^**^	0.849^**^	0.224	0.542^**^
**LDH**							1.000	0.664^**^	0.690^**^	0.466^*^
**AST**								1.000	0.569^**^	0.590^**^
**AST/CK**									1.000	0.105
**CK‐MB/H‐FABP**										1.000

*Note*: Spearman correlation coefficients (*r*).

Abbreviations: AST, aspartate aminotransferase; CK, creatine kinase; CK‐MB; creatine kinase myocardial band; H‐FABP, heart type fatty acid binding protein; LDH, lactate dehydrogenase; LMR, lymphocyte monocyte ratio; LYM, lymphocyte; WBC, white blood cell.

* *p* < 0.05.

** *p* < 0.01.

H‐FABP demonstrated a strong positive correlation with RBC (*r* = 0.888, *p* < 0.001), HGB (*r* = 0.825, *p* < 0.001), HCT (*r* = 0.808, *p* < 0.001) and PLT (*r* = 0.817, *p* < 0.001). MCH (*r* = −0.635, *p* < 0.001) and MCHC (*r* = −0.596, *p* = 0.002) were moderately negatively correlated with H‐FABP (Table 6).

**TABLE 6:** Spearman correlations between erythrocyte parameters and myocardial stress biomarkers in cattle.

Parameters	RBC	HGB	HCT	MCH	MCHC	PLT	H‐FABP	CK‐MB	CK	LDH	AST	AST/CK	CK‐MB/H‐FABP
**RBC**	1.000	0.944^**^	0.913^**^	**−0.598** ^**^	−0.585^**^	0.774^**^	**0.888** ^**^	**0.417** ^*^	0.344	0.658^**^	0.515^*^	0.649^**^	0.043
**HGB**		1.000	0.989^**^	**−0.391**	−0.649^**^	0.636^**^	**0.825** ^**^	0.414^*^	0.475^*^	0.589^**^	0.583^**^	0.598^**^	0.085
**HCT**			1.000	**−0.358**	−0.704^**^	0.601^**^	**0.808** ^**^	**0.428** ^*^	0.482^*^	**0.570** ^**^	**0.589** ^**^	**0.603** ^**^	**0.107**
**MCH**				1.000	0.385	−0.585^**^	**−0.635** ^**^	−0.285	0.032	−0.506^*^	−0.233	−0.513^*^	−0.055
**MCHC**					1.000	−0.307	**−0.596** ^**^	**−0.644** ^**^	**−0.626** ^**^	**−0.581** ^**^	**−0.717** ^**^	**−0.509** ^*^	**−0.468** ^*^
**PLT**						1.000	**0.817** ^**^	0.207	−0.003	0.568^**^	0.252	0.591^**^	−0.168

*Note*: Spearman correlation coefficients (*r*).

Abbreviations: AST, aspartate aminotransferase; CK, creatine kinase; CK‐MB; creatine kinase myocardial band; HCT, haematocrit; H‐FABP, heart type fatty acid binding protein; HGB, haemoglobin; LDH, lactate dehydrogenase; MCH, mean corpuscular haemoglobin; MCHC, mean corpuscular haemoglobin concentration; PLT, thrombocyte; RBC, red blood cell.

* *p* < 0.05.

** *p* < 0.01.

### 3.5 Diagnostic Performance of Biomarkers

**FIGURE 1:**
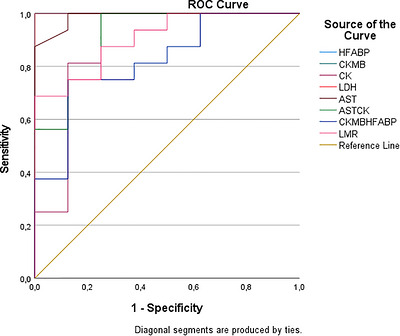
Receiver operating characteristic (ROC) curves of selected myocardial stress biomarkers and ratio‐derived indices for differentiation between BRDC‐affected and healthy cattle.

ROC analysis results are presented in Table 7 and Figure 1. H‐FABP, CK‐MB and LDH demonstrated apparent complete separation within this dataset (AUC = 1.000; 95% CI: 1.000–1.000); however, these findings likely reflect sample size–related overfitting rather than true diagnostic accuracy. AST (AUC = 0.988; 95% CI: 0.956–1.000), AST/CK (AUC = 0.914; 95% CI: 0.789–1.000) and LMR (AUC = 0.906; 95% CI: 0.789–1.000) demonstrated excellent discriminatory performance, whereas CK (AUC = 0.883; 95% CI: 0.704–1.000) demonstrated good discriminatory performance. An H‐FABP cut‐off value of 0.44 ng/mL yielded 100% sensitivity and specificity within the studied population, while an LMR cut‐off value of 32.5 showed 68.7% sensitivity and 100% specificity. Complete separation between groups for selected biomarkers should be interpreted cautiously, as perfect AUC values may reflect sample size limitations rather than true diagnostic performance. Accordingly, the ROC results should be interpreted as preliminary and hypothesis‐generating rather than definitive evidence of diagnostic performance, and external validation in independent cohorts is required to confirm these findings.

**TABLE 7:** Diagnostic performance of selected biomarkers and ratio‐derived indices for differentiating BRDC‐affected and healthy control cattle.

Parameter	AUC (95% CI)	Cut‐off	Sensitivity (%)	Specificity (%)	*p* value
H‐FABP (ng/mL)	1.000 (1.000–1.000)	> 0.44	100	100	< 0.001
CK‐MB (U/L)	1.000 (1.000–1.000)	> 189	100	100	< 0.001
CK (U/L)	0.883 (0.704–1.000)	> 123	100	75	< 0.001
LDH (U/L)	1.000 (1.000–1.000)	> 1259	100	100	< 0.001
AST (U/L)	0.988 (0.956–1.000)	> 68	100	87.5	< 0.001
AST/CK	0.914 (0.789–1.000)	> 0.2	100	75	< 0.001
CK‐MB/H‐FABP	0.820 (0.643–0.998)	> 543	75	87.5	0.001
LMR	0.906 (0.789–1.000)	> 32.5	68.7	100	< 0.001

Abbreviations: AST, aspartate aminotransferase; AUC, area under the receiver operating characteristic curve; CI, confidence interval; CK, creatine kinase; CK‐MB, creatine kinase myocardial band; H‐FABP, heart type fatty acid binding protein; LDH, lactate dehydrogenase; LMR, lymphocyte‐monocyte ratio.

### 3.6 Multivariable Regression Analysis

Multivariable linear regression analysis was performed to identify factors independently associated with H‐FABP concentrations. The overall model was statistically significant (*F* = 59.90, *p* < 0.001) and explained 85.1% of the variance in H‐FABP concentrations (*R* ^2^ = 0.851; adjusted *R* ^2^ = 0.837). RBC was independently associated with H‐FABP concentrations (standardized *β* = 1.033, *p* < 0.001). In contrast, LMR was not significantly associated with H‐FABP concentrations after adjustment for RBC (standardized *β* = −0.195, *p* = 0.087). No evidence of problematic multicollinearity was detected (VIF = 1.665; tolerance = 0.601 for both variables). Nevertheless, given the exploratory nature of the model and limited sample size, coefficient estimates should be interpreted cautiously and require confirmation in larger independent cohorts. Although LMR demonstrated a positive correlation with H‐FABP in univariable analysis (*r* = 0.441), its association was attenuated after adjustment for RBC, likely reflecting shared variance between the predictors rather than an independent biological effect.

## 4 Discussion

This study evaluated the relationship between myocardial stress biomarkers and haematological parameters in cattle with BRDC and explored the potential utility of ratio‐derived enzyme indices in characterizing tissue injury patterns. Significant elevations in H‐FABP, CK‐MB, LDH and AST were observed in BRDC‐affected cattle, accompanied by strong correlations between H‐FABP and vital parameters. These findings suggest that BRDC may involve cardiopulmonary stress mechanisms extending beyond primary pulmonary pathology (Anderson et al. 2017; Değirmençay 2023).

The strong positive correlation between H‐FABP and HR is consistent with previous veterinary and human studies demonstrating that H‐FABP increases rapidly following myocardial membrane injury and reflects ongoing myocardial stress (Değirmençay 2023; Lam et al. 2019; Netala et al. 2025; O'Donoghue et al. 2006; Yildiz et al. 2019). In the context of BRDC, hypoxemia, tachycardia and systemic inflammatory activation may increase myocardial oxygen demand while impairing oxygen delivery, thereby predisposing to cardiomyocyte stress and biomarker release (Anderson et al. 2017; Ceciliani et al. 2012). Accordingly, H‐FABP elevation in the present study likely reflects myocardial stress occurring in parallel with respiratory compromise. Although direct cardiac imaging or cardiac troponin measurements were not performed in the present study, the consistent associations observed between H‐FABP and physiological stress parameters such as HR and RR support the interpretation that myocardial stress may accompany systemic inflammatory responses in BRDC. Therefore, the observed biomarker alterations are most consistent with myocardial stress associated with systemic inflammatory and hypoxemic conditions rather than definitive evidence of primary myocardial necrosis.

Interpretation of CK‐MB, AST and LDH elevations requires caution, as these enzymes are not exclusively cardiac‐specific and may increase in skeletal muscle or hepatic injury during systemic inflammatory conditions (Dufour 1988; Wu et al. 2020). Although CK‐MB is relatively enriched in cardiac tissue, it may also increase in skeletal muscle injury under conditions of systemic stress. Importantly, in the absence of skeletal muscle–specific markers (e.g., myoglobin) and liver‐specific enzymes (e.g., GGT), the observed increases in CK, AST and LDH cannot be confidently attributed to myocardial sources. These biomarkers should therefore be interpreted as reflecting potential mixed tissue contributions rather than definitive evidence of cardiac injury. To address this limitation, ratio‐derived indices were evaluated. The AST/CK ratio has historically been used to aid differentiation between cardiac and skeletal muscle sources of enzyme elevation (Dufour 1988; Swain et al. 1993). The higher AST/CK ratio observed in BRDC cattle may reflect a relative shift in enzyme release patterns, potentially involving myocardial contribution; however, hepatic or skeletal muscle involvement cannot be excluded. Similarly, interpretation of the CK‐MB/H‐FABP ratio should consider the dual distribution of CK‐MB, which, although enriched in cardiac tissue, may also increase in skeletal muscle injury under systemic stress (Wu et al. 2020). Thus, rather than serving as definitive diagnostic tools, these ratios may contribute to a pattern‐based understanding of tissue injury dynamics in BRDC. Unlike a previous investigation focusing primarily on individual cardiac biomarkers (Değirmençay 2023), the present study adopts a pattern‐based approach integrating ratio‐derived indices and haematological inflammatory parameters to explore cardiopulmonary interactions in BRDC.

LMR was significantly elevated in BRDC‐affected cattle and showed a moderate positive correlation with H‐FABP. In human medicine, LMR has been associated with cardiovascular disease severity and prognosis, including acute coronary syndromes and heart failure (Chen et al. 2020; Kiris et al. 2017; Silva et al. 2015; Wang et al. 2017; Zhao et al. 2022). However, the biological interpretation of LMR in the present study differs fundamentally from that in human cardiovascular disease. In contrast to chronic cardiovascular conditions, where reduced LMR is often associated with adverse outcomes, the elevated LMR observed in BRDC likely reflects acute inflammatory responses characterized by dynamic changes in leukocyte subpopulations during infection. Therefore, direct comparisons between these contexts should be made with caution. Nevertheless, LMR should not be interpreted as a cardiac‐specific biomarker in the present context. Instead, it reflects alterations in leukocyte dynamics associated with systemic inflammatory activation. In BRDC, pulmonary inflammation and acute phase responses may alter leukocyte distribution and MON activity (Ceciliani et al. 2012). The association between LMR and H‐FABP likely represents the interaction between systemic inflammatory burden and myocardial stress rather than direct evidence of primary cardiac injury. This concept is further supported by evidence that MON‐driven inflammatory pathways are linked to adverse cardiovascular stress responses (Ren et al. 2017). Importantly, although LMR showed a significant positive correlation with H‐FABP in univariable analysis, this association was no longer statistically significant after adjustment for RBC in the multivariable model. The attenuation of the association and the apparent sign reversal of the regression coefficient likely reflect shared variance between inflammatory and erythrocyte‐related parameters rather than a true inverse biological relationship. Therefore, LMR should not be interpreted as an independent determinant of H‐FABP concentrations in the present study. Nevertheless, the observed univariable association suggests that LMR may still reflect aspects of systemic inflammatory burden accompanying myocardial stress biomarker elevation in BRDC and may serve as a readily accessible inflammatory indicator under field conditions.

A notable finding was the significant increase in RBC, HGB and HCT values in the BRDC group. In the absence of direct hydration assessment, these alterations are most plausibly explained by relative hemoconcentration secondary to reduced fluid intake, fever and increased insensible fluid losses associated with tachypnea. Hemoconcentration and stress‐associated erythrocyte changes have been described in inflammatory and systemic disease states (Weiss and Wardrop 2010). Increased HCT may elevate blood viscosity and thereby increase cardiac workload and myocardial oxygen demand. Within a hypoxemic and inflammatory environment, this hemodynamic alteration may further contribute to myocardial stress and biomarker elevation. Importantly, hemoconcentration itself may act as a confounding factor by non‐specifically increasing circulating concentrations of biomarkers, including H‐FABP, independent of true cellular injury. Therefore, part of the observed elevation in myocardial biomarkers may reflect concentration effects rather than increased release alone. Although plasma volume changes were not directly quantified in the present study, the influence of hemoconcentration on circulating biomarker concentrations cannot be completely excluded. Therefore, the contribution of hemoconcentration to biomarker elevation cannot be quantitatively distinguished in the present study. However, the presence of significant correlations between H‐FABP and physiological parameters such as HR and RR suggests that biomarker elevation is unlikely to be explained solely by hemoconcentration and may also reflect underlying cardiopulmonary stress. Therefore, the observed erythrocyte‐related changes are more likely to reflect physiological adaptation to hypoxemia and systemic stress rather than true primary erythrocytosis. Importantly, the strong correlation observed between RBC and H‐FABP suggests that erythrocyte‐related changes may not merely reflect hemoconcentration but could also be linked to physiological responses to hypoxemia and increased cardiopulmonary workload in BRDC. This interpretation is further supported by the multivariable regression analysis, in which RBC remained independently associated with H‐FABP concentrations after adjustment for LMR, suggesting that erythrocyte‐related changes may represent an important contributor to circulating H‐FABP concentrations in BRDC.

Cardiac troponins (cTnI and cTnT) are widely regarded as reference biomarkers of myocardial necrosis in both human and veterinary medicine (Anderson et al. 2017; O'Donoghue et al. 2006). However, H‐FABP is recognized as an early‐release biomarker that may increase in circulation within hours following myocardial membrane injury, often preceding detectable troponin elevations (Gerede et al. 2015). Therefore, H‐FABP may reflect early myocardial stress responses associated with hypoxemia and systemic inflammatory activation. In the present study, the observed associations between H‐FABP, physiological stress parameters and inflammatory indices support the interpretation that BRDC may involve cardiopulmonary stress affecting myocardial tissue. Nevertheless, these biomarker alterations should be interpreted as indicators of myocardial stress rather than definitive confirmation of primary myocardial necrosis.

**FIGURE 2:**
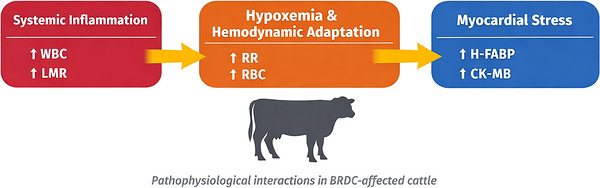
Proposed mechanistic framework illustrating the interaction between systemic inflammation, hypoxemia‐related haemodynamic adaptation and myocardial stress in cattle affected by bovine respiratory disease complex (BRDC). Increased inflammatory activity (e.g., WBC and LMR elevation) and physiological stress responses may contribute to cardiopulmonary strain and elevation of myocardial stress biomarkers such as H‐FABP and CK‐MB.

Taken together, the combination of biochemical (H‐FABP, CK‐MB, LDH and AST), haematological (LMR) and ratio‐derived (AST/CK, CK‐MB/H‐FABP) indicators provides a multifaceted perspective on tissue injury patterns in BRDC. Conceptually, these findings support a cardiopulmonary stress model in which systemic inflammatory activation and hypoxemia‐related physiological adaptation interact to promote myocardial stress biomarker release in BRDC‐affected cattle (Figure 2). Such an integrated approach may enhance understanding of cardiopulmonary interactions in complex inflammatory conditions.

From a clinical perspective, these findings suggest that myocardial stress may represent an underrecognized component of the systemic pathophysiology of BRDC. In field conditions where advanced cardiac diagnostics are rarely available, the combined interpretation of myocardial stress biomarkers, haematological inflammatory indices and physiological parameters may offer a practical framework for identifying cardiopulmonary stress responses in affected cattle. Such an integrated biomarker approach may therefore contribute to improved clinical interpretation of disease severity and systemic involvement in BRDC.

Several limitations should be acknowledged. The relatively small sample size may limit generalizability and increase the risk of overestimating diagnostic performance in ROC analyses. In addition, the high diagnostic performance observed in ROC analyses should be interpreted cautiously, as small sample size may lead to optimistic estimations of sensitivity and specificity, including apparently perfect diagnostic discrimination in some biomarkers. The cross‐sectional design of the study precludes inference of causality between systemic inflammatory burden and myocardial biomarker elevation. The absence of echocardiographic or histopathological confirmation precludes definitive verification of myocardial injury. Hydration status was not directly assessed, limiting interpretation of erythrocyte‐related findings. Furthermore, given the limited sample size, the multivariable regression model should be interpreted as exploratory and requires validation in larger cohorts. Future studies incorporating larger cohorts, longitudinal designs, advanced imaging modalities and comprehensive hydration assessment would provide more robust evidence regarding the cardiopulmonary interactions observed in BRDC.

Overall, the findings support the concept that BRDC should not be viewed solely as a respiratory disorder but rather as a condition involving integrated cardiopulmonary stress, where systemic inflammation and hypoxemia may contribute to elevations in myocardial stress biomarkers. Collectively, these observations suggest that cardiopulmonary interactions may represent an underrecognized component of BRDC pathophysiology and that integrated biomarker assessment could provide a potentially useful exploratory framework for evaluating cardiopulmonary stress responses in affected cattle under field conditions.

In conclusion, this exploratory study indicates that BRDC in cattle may be accompanied by elevations in circulating myocardial stress biomarkers, particularly H‐FABP and CK‐MB, in parallel with systemic inflammatory and haematological alterations. Significant associations between H‐FABP and vital parameters, together with independent association of RBC with H‐FABP concentrations in multivariable analysis, suggest an interaction between erythrocyte‐related changes, inflammatory burden and myocardial stress responses. Ratio‐derived enzyme indices (AST/CK and CK‐MB/H‐FABP) and haematological parameters such as LMR appear to contribute to a pattern‐based interpretation of tissue injury dynamics rather than serving as definitive markers of myocardial necrosis. However, these findings should be interpreted as preliminary given the exploratory design, limited sample size and absence of external validation. While integrated evaluation of biochemical and haematological indicators may offer a potentially useful exploratory framework for characterizing cardiopulmonary stress patterns in BRDC, further studies with larger cohorts, longitudinal designs and validated cardiac reference markers are required to confirm the clinical relevance and diagnostic utility of these observations.

## Author Contributions

**Şükrü Değirmençay**: conceptualization, data curation, formal analysis, investigation, methodology, resources, software, supervision, validation, visualization, writing – original draft, writing – review and editing. **Reyhane Bayat**: data curation, formal analysis, investigation, methodology, writing – original draft, writing – review and editing.

## Funding

The authors have nothing to report.

## Ethics Statement

This research was conducted in compliance with the ethical guidelines approved by Atatürk University (protocol number: 2025/08, decision number: 147).

## Consent

Written informed consent was obtained from the owner of each animal prior to sample collection.

## Conflicts of Interest

The authors declare no conflicts of interest.

## Supporting information



Supporting Information 1: vms371096‐sup‐0001‐SuppData.pdf

## Data Availability

The data supporting the findings of this study are available within the article and it's Supporting Information (Table S1).
