# Supplementary material for: Myocardial Stress Biomarkers and Inflammatory Indices in Cattle With Bovine Respiratory Disease Complex
Source: Vet Med Sci. 2026 Jul 20;12(4):e71096. doi: 10.1002/vms3.71096 (PMC13384338; doi:10.1002/vms3.71096)
Supplement: Supplementary file 1 — Supporting Information 1: vms371096‐sup‐0001‐SuppData.pdf [file VMS3-12-e71096-s001.pdf]

Myocardial Stress Biomarkers and Inflammatory Indices in Cattle with Bovine Respiratory Disease Complex

Description:
This supplementary dataset contains the individual-level clinical, hematological, biochemical, and biomarker measurements used for statistical analyses in the present study. Animals were classified as healthy controls (Group = 0) or BRDC-affected cattle (Group = 1).

Group codes:
0 = Healthy control
1 = BRDC

Supplementary Table S1: Individual-level clinical, haematological, and biochemical data from healthy control and BRDC-affected cattle included in the study.

**No**	**WBC**	**LYM**	**MON**	**NEU**	**EOS**	**BAS**	**NLR**	**LMR**	**RBC**	**HGB**	**HCT**	**MCV**	**MCH**	**MCHC**	**PLT**	**H-FABP**	**CK-MB**	**CK**	**AST**	**LDH**	**AST/CK**	**CK-MB/H-FABP**	**RT**	**RR**	**HR**	**Clinical score**
0	4,59	2,05	0,17	2,31	0,05	0	1,126829	12,05882	8,2	9,7	27,37	33	11,9	35,5	498	0,41	169,7	115	14	336	0,121739	413,9024	39	28	80	0
0	7,62	6,29	0,68	0,52	0,1	0,03	0,082671	9,25	5,48	5,4	15,73	29	9,9	34,6	174	0,27	189	123	19	487	0,154472	700	39,1	24	76	0
0	6,55	3,67	0,29	2,54	0,05	0	0,692098	12,65517	5,65	6,2	16,97	30	10,9	36,4	278	0,37	179,6	122	20	505	0,163934	485,4054	39,1	36	78	0
0	5,67	1,87	0,07	3,63	0,09	0,01	1,941176	26,71429	6,81	8,3	23,48	34	12,1	35,2	211	0,33	59,9	344	68	638	0,197674	181,5152	38,9	56	80	0
0	4,63	2,47	0,13	1,83	0,2	0,01	0,740891	19	5,66	5,4	16,17	29	9,5	33,4	310	0,39	118,4	82	28	493	0,341463	303,5897	38,4	32	68	0
0	8,33	4,03	0,24	3,9	0,15	0,01	0,967742	16,79167	5,52	6,5	18,09	33	11,8	36	400	0,44	94,9	59	10	278	0,169492	215,6818	39,3	28	60	2
0	8,17	2,6	0,08	5,34	0,15	0,01	2,053846	32,5	5,84	6,7	19,25	33	11,4	34,6	361	0,22	93,7	70	14	336	0,2	425,9091	39,2	36	88	0
0	7,36	4,36	0,34	2,54	0,11	0	0,582569	12,82353	5,24	7,9	23,82	45	15,2	33,3	239	0,32	173,9	191	71	1259	0,371728	543,4375	38,8	28	88	0
1	10,2	5,66	0,35	4,11	0,05	0,03	0,726148	16,17143	10,6	10,8	35,18	33	10,1	30,6	482	0,71	435	325	127,7	2778,3	0,392923	612,6761	39,95	44	144	12
1	6,95	3,98	0,14	2,79	0,02	0,02	0,701005	28,42857	10,73	10,5	34,08	32	9,8	30,8	589	0,63	561	360	139,3	2646,3	0,386944	890,4762	40,16	52	140	12
1	7,01	4,46	0,06	2,45	0,02	0,02	0,549327	74,33333	11,6	11,6	38,9	34	10	29,8	465	0,65	374	249	89,4	2093,4	0,359036	575,3846	39,25	48	160	8
1	6,88	4,4	0,1	2,24	0,07	0,07	0,509091	44	12,6	12,4	39,6	31	9,9	31,4	749	0,74	333	232	112,6	2792,8	0,485345	450	39,04	44	132	10
1	9,74	5,27	0,07	4,32	0,05	0,03	0,819734	75,28571	12,52	11,8	38,49	31	9,4	30,5	568	0,66	400	311	99	2720,3	0,318328	606,0606	39,12	52	144	10
1	10,77	4,43	0,2	6,03	0,07	0,04	1,361174	22,15	10,2	10,6	35,63	35	10,4	29,8	419	0,58	448	333	109,1	2570,8	0,327628	772,4138	40,7	52	152	12
1	5,45	3,62	0,08	1,63	0,1	0,02	0,450276	45,25	10,47	11,4	38,78	37	10,9	29,4	150	0,49	505	372	131,8	2572,3	0,354301	1030,612	38,86	36	120	8
1	11,49	5,87	0,08	5,4	0,08	0,06	0,919932	73,375	13,58	12,9	41,41	30	9,5	31,2	731	0,77	252	174	70,5	2212,4	0,405172	327,2727	39,67	44	184	12
1	12,36	6,25	0,33	5,72	0,04	0,02	0,9152	18,93939	12,89	12,6	41,4	32	9,8	30,4	628	0,76	293	295	125,6	2150,3	0,425763	385,5263	40,1	44	167	12
1	11,41	5,23	0,07	6	0,06	0,04	1,147228	74,71429	10,85	11,5	36,83	34	10,6	31,3	405	0,53	464	367	122,2	2585,1	0,33297	875,4717	40,2	52	161	12
1	7,16	4,39	0,09	2,7	0	0	0,615034	48,77778	11,12	11,1	35,71	32	10	31,1	618	0,6	583	283	109,3	2613	0,386219	971,6667	40,48	48	142	8
1	7,63	4,4	0,09	3,02	0,07	0,05	0,686364	48,88889	11,85	12,5	41,32	35	10,5	30,2	439	0,62	346	372	139	2087	0,373656	558,0645	39,15	48	162	8

1	8,92	4,39	0,08	4,28	0,12	0,05	0,974943	54,875	11,11	11,1	35,71	32	10	31,1	460	0,74	449	167	71	2640,8	0,42515	606,7568	40,2	44	151	12
1	7,94	4,96	0,11	2,73	0,09	0,05	0,550403	45,09091	12,04	12,6	41,41	34	10,5	30,5	558	0,69	392	341	110,2	2612,4	0,323167	568,1159	39,07	50	148	6
1	6,11	4,45	0,1	1,46	0,08	0,02	0,32809	44,5	11,01	11,5	37,32	34	10,5	30,9	199	0,48	473	331	118,9	2605	0,359215	985,4167	38,89	36	106	6
1	6,89	3,58	0,18	3,05	0,05	0,03	0,851955	19,88889	12,45	12	39,77	32	9,6	30,1	846	0,75	311	181	89	2793	0,491713	414,6667	38,98	36	139	10

WBC, white blood cell count; LYM, lymphocyte count; MON, monocyte count; NEU, neutrophil count; EOS, eosinophil count; BAS, basophil count; NLR, neutrophil-to-lymphocyte ratio; LMR, lymphocyte-to-monocyte ratio; RBC, red blood cell count; HGB, hemoglobin; HCT, hematocrit; MCV, mean corpuscular volume; MCH, mean corpuscular hemoglobin; MCHC, mean corpuscular hemoglobin concentration; PLT, platelet count; H-FABP, heart-type fatty acid binding protein; CK-MB, creatine kinase-MB; CK, creatine kinase; AST, aspartate aminotransferase; LDH, lactate dehydrogenase; RT, rectal temperature; RR, respiratory rate; HR, heart rate.
